# Transactional Pathways from Early Externalizing Behavior to Adolescent Delinquency: The Roles of Father Engagement and Parenting Aggravation

**DOI:** 10.1007/s10964-026-02402-0

**Published:** 2026-08-19

**Authors:** Abigail J Anderson, Christopher C Henrich, Sylvie Mrug

**Affiliations:** University of Alabama at Birmingham, Birmingham, USA

**Keywords:** Delinquency, Father aggravation in parenting, Father engagement, Child externalizing behavior problems, Developmental cascade framework, Bidirectional relationships, Indirect effects

## Abstract

Although fathers play a critical role in their children’s outcomes, the role of fathers in the developmental pathways of delinquency remains understudied. Guided by a developmental cascade framework, this study examined prospective, bidirectional associations among child externalizing behaviors, father engagement, and father aggravation in parenting across childhood in predicting adolescent delinquency. Using data from the Future of Families and Child Wellbeing Study (*N* = 3,296; 47% female; 47% Black Non-Hispanic; 22% White Non-Hispanic; 27% Hispanic), a cross-lagged panel model was estimated to test the reciprocal associations and indirect effects between father characteristics and early child externalizing behavior problems across four waves of assessment (average child age three, five, nine, and 15 years old). Results indicated limited evidence of bidirectional associations among father engagement, father aggravation, and child externalizing behaviors. Early child externalizing behavior was the most consistent predictor of adolescent delinquency. One small, unexpected association emerged in which higher father aggravation at age 3 predicted greater father engagement at age 5. No indirect effects were found. These findings underscore early externalizing behavior as a key developmental precursor of delinquency and suggest that fathers’ early engagement and aggravation in parenting play a limited role in the development of adolescent delinquency when considered within broader family processes.

**Supplementary Information:**

The online version contains supplementary material available at https://doi.org/10.1007/s10964-026-02402-0.

## Introduction

Engagement in delinquent behaviors, such as theft, vandalism, and aggression, is one of the central features of adolescence (Barbot & Hunter, 2012), with rates typically peaking around 15 years of age, though variations in type and context have been noted (Willoughby et al., 2021). Despite declines in recent years (Bureau of Justice Statistics, 2024), delinquency continues to pose a significant public health concern due to the long-term negative consequences for youth and society, as reflected in the estimated 424,300 juvenile arrests reported in 2020 (Puzzanchera et al., 2022). These adverse consequences typically manifest as lower educational achievement, greater likelihood of unemployment, incarceration, and poorer mental and physical health (Carter, 2019; Drury et al., 2020; Kim et al., 2020). A central challenge for prevention research is identifying early developmental processes that increase risk for later delinquency and clarifying how these processes unfold across childhood within the family context. This study addresses this challenge by examining longitudinal, reciprocal associations among early child behavior problems, father engagement, and fathers’ aggravation in parenting, and by testing how these processes indirectly contribute to adolescent delinquency.

### Early Externalizing Behaviors as Precursors to Delinquency

One of the most salient predictors of juvenile delinquency is externalizing behavior problems in early childhood (Assink et al., 2015). Externalizing behavior problems refer to a broad class of behavioral difficulties characterized by outwardly directed actions, such as aggression, attention problems, and noncompliance (Achenbach et al., 2016). Although these externalizing behavior problems typically decline as children age (Jia-Richards et al., 2025), for some youth they remain high or even escalate. From a developmental perspective, these persistently elevated behaviors are particularly important to identify because they may signal a trajectory toward more serious and chronic forms of delinquency (Moffitt & Caspi, 2001), as well as related long-term outcomes, such as decreased academic performance, poor mental health, impaired social competency, and substance use problems (Gargano et al., 2018). Although externalizing behaviors and delinquency share conceptual overlap, such as rule-breaking and aggression, they are not interchangeable. Delinquency represents a narrower, legally defined subset of the broader externalizing spectrum that can include behaviors such as vandalism, physical assault, and substance use (Isen et al., 2022). By contrast, early externalizing behaviors reflect broader regulatory difficulties, such as temper tantrums and hyperactivity (Achenbach et al., 2016), that may set the stage for later delinquency (Thompson et al., 2011). This distinction underscores the importance of examining early externalizing behaviors as developmental precursors for later delinquency.

### Fathers’ Roles in the Development of Child and Adolescent Behavior

As the primary socializing context in childhood and adolescence, family represents a key environment to target for delinquency prevention (Climent-Galarza et al., 2022). In family research, the role of fathers has historically received less attention compared to mothers, despite the evidence that paternal characteristics uniquely contribute to children’s cognitive, emotional, and behavioral outcomes (Sarkadi et al., 2008), including the emergence and maintenance of adolescent delinquency (Cobb-Clark & Tekin, 2011).

Two important aspects in the role of fathers are engagement and aggravation in parenting. Father engagement typically refers to direct interactions between fathers and children, with the nature of these interactions changing across development in response to children’s evolving needs (Lamb, 2000). Higher levels of father engagement are associated with positive child outcomes (Carlson & McLanahan, 2010), such as better behavior regulation (Easterbrooks et al., 2014), academic success (Jeynes, 2015), and reduced externalizing behaviors and delinquency (Dwyer Emory, 2018). Fathers who use more frequent positive engagement activities, which include age-appropriate interactions meant to promote positive development, such as helping the child with homework, singing songs, or playing a sport together (Temmen & Crocket, 2021), have children with less externalizing behaviors (Slaughter & Nagoshi, 2020).

Father aggravation in parenting refers to the anxiety, stress, and strain that fathers experience in the parenting role (Fagan & Lee, 2014). More specifically, aggravation in parenting, often referred to as parenting stress, results from the discrepancy between the demands of parenting and parents’ available resources (Deater-Deckard & Panneton, 2017), often marked by parents feeling that being a parent is harder than they thought it would be and feelings of being trapped or exhausted from these responsibilities. This construct is conceptually distinct from stress experienced in other domains of life and is strongly related to parenting behaviors (Deater-Deckard, 1998). When parents experience stress related to the parenting role, they may respond less effectively or less positively to their children’s needs (Ward & Lee, 2020). The link between aggravation in parenting and early child externalizing behavior problems has been well established in the literature (e.g., Stone et al., 2016), with aggravation in parenting contributing to poorer developmental outcomes (Bronte-Tinkew et al., 2010). Although relatively little research has examined adolescent outcomes such as delinquency (Kochanova et al., 2022), externalizing behaviors are a well-established precursor to delinquent behavior, suggesting that similar associations may extend to delinquency in adolescence.

### Transactional and Bidirectional Family Processes

Associations between child externalizing behaviors and paternal characteristics are dynamic and often bidirectional. That is, parents mold their children’s behaviors, but children also shape their parents’ behaviors through child evocative effects (Leve & Cichetti, 2016), which may then spill over into delinquency. Despite theoretical emphasis on transactional processes, most existing research on father engagement, parenting aggravation, and child externalizing behavior problems has focused on unidirectional associations.

In general, fathers’ engagement is associated with fewer externalizing behaviors in children and less delinquency in adolescence (Dwyer Emory, 2018; Yoder et al., 2016), suggesting the importance of father engagement in development. Less research has focused on the opposite direction, in that children may shape their father’s level of engagement. From the perspective of child evocative effects, elevated externalizing behaviors may reduce the opportunity for positive engagement activities by increasing conflict and parenting aggravation, and in turn, low engagement may increase the likelihood of externalizing and delinquent behaviors over time. Despite theoretical expectations of bidirectional influences over time, empirical findings have been limited and mixed. In a model of bidirectional effects, one study found only child-driven effects in early development, with father engagement not related to subsequent child externalizing problem behaviors across early to middle childhood (Flouri et al., 2016). Similarly, one study did not find evidence of a prospective bidirectional relationship between child externalizing behaviors and father engagement over the course of early childhood to adolescence using data from the Future of Families and Child Wellbeing study (Anderson et al., 2024).

Although not studied in fathers, evidence supports both the effect of externalizing behaviors on aggravation in parenting (McDaniel & Radesky, 2020), as well as the effect of parenting aggravation on externalizing behaviors (Pei et al., 2019). Thus, children’s externalizing behaviors may lead to greater parenting aggravation in their fathers through increased caregiving strain and difficulty managing child behavior, consistent with evocative effects. In turn, elevated parenting aggravation may undermine effective parenting practices and contribute to increases in child externalizing behaviors over time. Although few studies have examined these processes specifically among fathers, emerging evidence from maternal samples supports these reciprocal relationships between parenting aggravation and child behavior problems (Jiang et al., 2023), highlighting the need for father-focused longitudinal research. Together, these findings point to the need for more longitudinal research to test the transactional, bidirectional pathways between father characteristics and child outcomes across childhood and adolescence.

Beyond their associations with child behavior, father engagement and father aggravation in parenting may also be dynamically related to one another, highlighting the importance of considering how different dimensions of paternal functioning operate in tandem across development. Aggravation in parenting may manifest as frustration, anger, and exhaustion (Yu & Singh, 2012), which may strain the parent-child relationship and reduce opportunities for positive father-child interactions, ultimately contributing to lower levels of father engagement over time (Ward & Lee, 2020). Conversely, low engagement may increase parenting demands concentrated in fewer interactions, potentially heightening feelings of frustration and aggravation during those interactions. Considering these relationships during infancy, one study confirmed that adolescent fathers’ aggravation in parenting was negatively related to their involvement in caregiving (Fagan et al., 2007). However, these associations remain understudied, particularly in longitudinal studies.

### Developmental Cascade Framework

A developmental cascade framework (e.g., Masten & Cicchetti, 2010) provides a useful conceptual lens for addressing these questions. Developmental cascade models describe how early risk in one domain may spill over into other domains over time, amplifying vulnerabilities and shaping later outcomes, including adolescent delinquency (e.g., Schneider & West, 2025). Within a developmental cascade framework, behaviors are viewed not only as the product of accumulating influences over time but also as factors that may trigger a cascade of developmental consequences across domains (Schneider & West, 2025). Importantly, cascade models allow for both direct and indirect effects, as well as unidirectional and bidirectional pathways, while accounting for the stability and concurrent associations of constructs across development (Masten & Cicchetti, 2010). Unlike models focused solely on concurrent or bidirectional and transactional parent-child effects, developmental cascade models emphasize how risks originating in one domain may accumulate and spread across multiple domains over time, producing indirect effects on later adjustment. Using this perspective, early child externalizing behavior problems may contribute to lower father engagement and greater parenting aggravation, which in turn may reinforce child externalizing behaviors and set the stage for delinquent behavior in adolescence. Applying this framework to father engagement and aggravation in parenting offers a theoretically grounded approach for clarifying how early family processes may cumulatively contribute to adolescent delinquency.

### Sex Differences

When considering the relationships between father engagement, father aggravation in parenting, child externalizing behaviors, and teen delinquency, consideration of potential sex differences is warranted. Sex differences in delinquency are one of the most well-replicated findings in the literature (Fairchild & Smaragdi, 2018), with the gender gap being found across time, cultures, and data sources (Choy et al., 2017). In earlier development, pre-school-aged boys are more likely to engage in externalizing behaviors than girls (Paz et al., 2021). Although not well studied, fathers tend to be more engaged with male than female children (Ogg & Anthony, 2019). However, a systematic review found no differences in parenting stress by child sex across studies that included mothers, fathers, and parents irrespective of gender (Fang et al., 2024).

Despite evidence of sex differences in child externalizing behavior problems, delinquency, and father engagement, research has rarely examined whether the pathways connecting fathering processes and child behavioral outcomes operate differently for boys and girls. Although not considering fathers specifically, one study found that parental involvement mediated the relationship between teen delinquency and adult crime for females, but not for males (Walters, 2013). By contrast, other research suggests that fathers may exert a larger influence on males’ externalizing behaviors compared to females (Gryczkowski et al., 2010). However, one study did not find sex differences in the links between father engagement, child externalizing behavior problems, and teen delinquency (Anderson et al., 2024), in line with research indicating that adolescent gender did not moderate the relationship between fathers’ positive engagement activities and adolescent externalizing behaviors (Temmen & Crocket, 2021). Additionally, one meta-analysis found the relationship between aggravation in parenting and externalizing behavior problems was stronger in samples with a higher percentage of males (Barroso et al., 2018). Taken together, prior findings provide inconsistent evidence regarding potential moderating effects of child sex in the associations among paternal variables, child externalizing behaviors, and delinquency, underscoring the need for further investigation.

### Role of Mothers

When studying fathers, the role of mothers needs to be considered due to their significant role in parenting and the interdependence between mothers and fathers (Scott et al., 2018). From a Family Systems Theoretical Perspective (Cox & Paley, 1997), families operate as a system of individuals and relationships, with members of the family being interdependent and affecting one another. Given this interdependence, studying fathers and father-child relationships in isolation is not recommended (Wang et al., 2022). Accordingly, accounting for maternal variables when examining fathers’ engagement and aggravation will help distinguish father-specific associations from broader family processes that jointly influence parenting and child development.

## Current Study

**Fig. 1:**
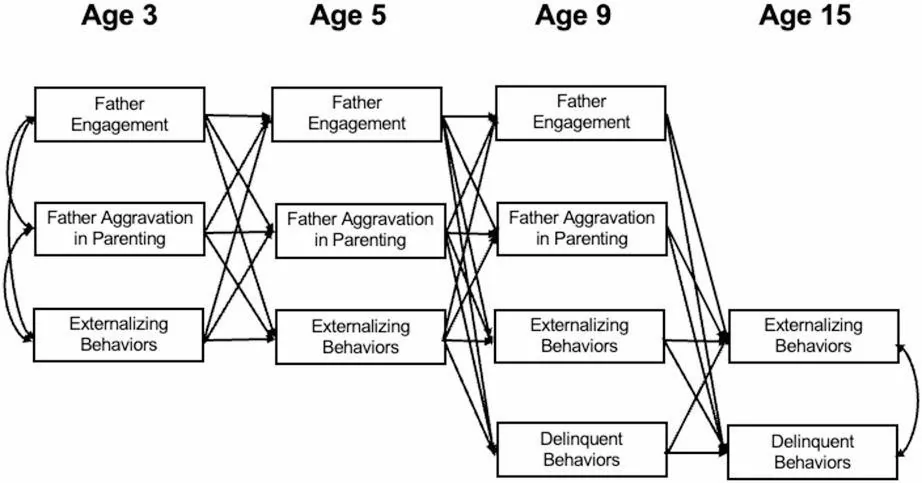
Hypothesized path model of the reciprocal effects between father engagement, father aggravation in parenting, and early child behaviors in predicting subsequent delinquency. All paths are adjusted for the covariates of mother engagement with the child, mother aggravation in parenting, parental relationship status, father education, father race, family poverty ratio, and child sex

Guided by the developmental cascade framework (Masten & Cicchetti, 2010), this study examined prospective effects between child externalizing problem behaviors, fathers’ engagement, and fathers’ aggravation in parenting across childhood, as well as their links to adolescent delinquency. Using four waves of data collected when children were three, five, nine, and 15 years old, the study tested reciprocal associations among these constructs while accounting for their stability over time and concurrent associations. As shown in Fig. 1, higher father engagement at age 3 was expected to be related to lower paternal aggravation in parenting and child externalizing behavior problems at age 5, whereas greater aggravation in parenting at age 3 was expected to be related to lower subsequent engagement and greater child behavior problems at age 5. Additionally, in line with child evocative effects, child externalizing behaviors at age 3 were expected to be related to higher subsequent father aggravation in parenting and lower father engagement at age 5. The same relationships among these constructs were expected between ages 5 and 9. Finally, greater paternal aggravation in parenting and lower father engagement at child age 9 were expected to be related to greater adolescent externalizing behaviors and delinquency at age 15.

In terms of indirect effects, it was hypothesized that father aggravation in parenting at age 3 would be indirectly related to later delinquency at age 15 through greater externalizing behaviors at ages 5 and 9, as well as lower father engagement at ages 5 and 9. It was also expected that child externalizing behavior problems at age 3 would be associated with higher father aggravation at age 5, which would in turn be related to lower levels of father engagement at age 9. Lastly, it was hypothesized that lower father engagement at age 5 would be indirectly related to teen delinquency at age 15 through greater child externalizing behavior problems at age 9. Additionally, this study examined child sex as a moderator of the prospective associations among father engagement, aggravation in parenting, child externalizing behaviors, and adolescent delinquency. Although some evidence suggests that pathways involving externalizing behaviors and delinquency may be stronger among boys than girls, findings regarding sex differences in the links between father engagement, father aggravation in parenting, child externalizing, and delinquency remain inconsistent. Consequently, moderation analyses were considered exploratory. Finally, given the significant role of mothers in parenting and the interdependence between mothers and fathers, maternal factors were included as time-varying covariates in all models to better isolate associations specific to fathers, with parent relationship status included to control for variations in family structure.

## Method

### Participants and Procedures

The Future of Families and Child-Wellbeing Study is a longitudinal, birth cohort study that followed nearly 5,000 children from 20 large, urban US cities beginning in 1998. The initial recruitment and data collection occurred at the time of the focal child’s birth, where families were randomly and sequentially sampled within hospitals to increase responsivity (FFCWS, n.d.; Kennedy & Gelman, 2018; Reichman et al., 2001). In order to learn more about contemporary family compositions, the FFCWS purposefully oversampled births to unwed parents, with parents not required to be married, cohabiting, or in an ongoing romantic relationship. Considering the roughly 4,700 families in the sample, the study design utilized a three-to-one sample of nonmarital to marital births, resulting in 3,600 unwed couples and 1,100 married couples. FFCWS was designed to investigate the environmental and social factors that shape the development of vulnerable children, thus oversampling Black, Hispanic, and low-income families. Throughout the study, approximately 50% of the families identified as Black non-Hispanic, 27% as Hispanic, and 22% as White non-Hispanic. The study then followed up with participating families at child age one year (*N* = 4,457), three years (*N* = 4,365), five years (*N* = 4,295), nine years (*N* = 3,813), 15 years (*N* = 3,595), and 22 years (*N* = 3,242). Data from ages three, five, nine, and 15 (Fig. 1) were utilized in this project.

In the Future of Families and Child Well-Being Study, both mothers and fathers completed separate parent interviews and reported on select study variables, such as engagement and aggravation in parenting. However, the parent designated as primary caregiver completed additional assessments, which included the child externalizing measures utilized in the current study, as they were deemed the person who had the most responsibility for the child. At age 3, 99% of respondents who completed the primary caregiver survey (*n* = 3,129) were the biological mothers. Given the low number of primary caregivers that were not biological mothers, including biological fathers, grandparents, other relatives, and foster parents, and to further control for confounding factors, primary caregivers who were not the biological mother were removed, with the term *mother* used to refer to primary-caregiving mothers.

Given that father engagement and father aggravation were central focal constructs in the longitudinal models, the analytic sample was restricted to families with at least one valid father report across the included waves, with fathers needing to report having seen the child within the past 30 days. The analytic sample for the current study comprised 3,296 children (48% female, 52% Male; 47% Black non-Hispanic; 27% Hispanic; 22% White non-Hispanic) and their families.

### Measures

#### Child Externalizing Behavior Problems

During all included time points, the mother reported on the focal child’s externalizing behaviors using the Child Behavior Checklist (Achenbach, 1992). For purposes of studying only externalizing behaviors specifically, different CBCL subscales were administered across developmental periods to capture age-appropriate manifestations of externalizing behavior. For this project, the Aggressive, Delinquent, and Rule-Breaking behavior subscales were used to measure child externalizing problem behaviors, which include behaviors like tantrums, sudden changes in mood or feelings, threatening people, being unusually loud, lying, cheating, vandalizing, and setting fires. Mothers indicated whether each statement was not true (0), sometimes or somewhat true (1), or very true or often true (2). Given the appropriateness of certain behaviors across development, the used subscales differ across waves. At age 3, 19 items from the Aggressive subscale were used. At ages 5 and 9, 30 items were used from both the Aggressive and Delinquent Subscales. At age 15, 20-items from the Aggressive and Rule-Breaking behavior subscales were used. At each time point, all items from the included subscales were averaged to create a composite child externalizing behavior problem score, with higher scores indicating more behavior problems. The Child Behavior Checklist has shown high convergent validity with other measures of children’s externalizing behavior problems (Achenbach & Rescorla, 2000; Rescorla, 2005). The current sample showed good reliability (α = 0.87-0.90).

#### Father Engagement

Father engagement was measured with 13 items at child age 3 and 8 items at age 5. Fathers reported the number of days per week that they engaged in certain developmentally appropriate behaviors with their children, such as playing games like “peek-a-boo”, singing songs, reading stories, playing inside with toys, hugging or showing affection, and putting the child to bed. Previous studies utilizing the FFCWs data have used these scales and evidenced adequate reliability (α = 0.81) (e.g., Lee, 2021), with studies using confirmatory factor analysis to support the structure (Anderson et al., 2024). At age 9, fathers provided information on father engagement with 10 items measuring how often in the past month the father and child did certain activities together, such as household chores, outdoor activities, and homework. Items were measured on a 4-point scale ranging from 0 (never) to 4 (every day). In previous studies of the FFCWs data, the Father Involvement Scale showed good reliability (α = 0.92) (e.g., Dwyer Emory, 2018), with factor structure supported by confirmatory factor analysis (Anderson et al., 2024). At each time point, the included questions were averaged to create a composite father engagement score, allowing for comparability of the construct across time despite differences in item composition. The measures of father engagement across waves demonstrated acceptable to good reliability (α = 0.75-0.86).

#### Father Aggravation in Parenting

At child ages 3, 5, and 9, father aggravation in parenting was measured using a four-item scale from the Parenting Stress Index (PSI) (Abidin, 1983). Fathers were asked if being a parent is harder than they thought it would be, if they feel trapped by their responsibilities as a parent, if they find that taking care of their child is much more work than pleasure, and if they often feel tired, worn out, or exhausted from raising a family. Items were measured on a 4-point scale from strongly agree (1) to strongly disagree (4). Items were reverse coded and summed, with higher scores indicating greater aggravation in parenting. Previous literature using this 4-item short form in the FFCWS data showed acceptable reliability (α = 0.77) (e.g., Bronte-Tinkew et al., 2010). Previous research also demonstrated validity of this scale (Annunziato et al., 2007), including a negative relationship with father engagement (Bronte-Tinkew et al., 2010). In the current study, father aggravation in parenting had adequate reliability (α = 0.61-0.67).

#### Delinquency

During the age 9 and 15 interviews, children self-reported their engagement in delinquency. Questions were adopted from measures in the National Longitudinal Study of Adolescent to Adult Health (Harris & Udry, 2018). The child reported their engagement in different behaviors in the past year, such as “gotten into a serious physical fight”, “gone into a house or building to steal something”, and “driven a car without its owner’s permission”. At age 9, children reported whether they engaged in 17 different delinquent behaviors (0 = No, 1 = Yes) which were modeled after the “Things that you have done” scale (Maumary-Gremaud, 2000), with previous research evidencing acceptable reliability using the FFCWS sample (α = 0.70) (e.g., Cheung & Huang, 2024). At age 15, teens self-reported whether they engaged in 13 different delinquent behaviors in the past year (0 = No, 1 = Yes). Items were summed, with higher scores indicating engagement in a wider range of delinquent behaviors. Acceptable reliability of this scale has been shown in previous literature (α = 0.72) (e.g., Gajos et al., 2023), and validity was supported by positive correlations with early conduct problems (Awada et al., 2020). In the current sample, delinquency evidenced acceptable reliability at both waves (α = 0.70-0.72).

#### Covariates

Analyses controlled for primary caregiving mothers’ self-reported engagement with the focal child and primary caregiving mothers’ self-reported aggravation in parenting at ages 3, 5, and 9, ensuring that the effects that may be attributed to fathers are not conflated with or overshadowed by mothers’ effects. Utilizing the same measures used for fathers, mother engagement has indicated acceptable reliability in previous research (α = 0.68) (Yang et al., 2024), and adequate reliability in the current study (α = 0.67-0.71). Mirroring the items used to measure father aggravation in parenting, at ages 3, 5, and 9, mother aggravation in parenting showed low reliability in the current study (α = 0.64-0.66).

Further, Parental Relationship Status (0 = Not together, 1 = Together) at ages 3, 5, and 9, Father Education (0 = High School and Below, 1 = Higher Education) at age 3, Family Income to Poverty Ratio (family household income divided by the poverty threshold) at age 3, Child sex (Male = 0, Female = 1), and Father Race (dummy variables for African American and Hispanic, with White non-Hispanic as the reference group) were included in the analyses as covariates.

### Analytic Strategy

Preliminary analyses were conducted using Mplus V8.8, including means, standard deviations, and bivariate correlations. Missing data analysis was used to test whether demographic or outcome variables differed between those retained over time versus those not retained using logistic regression. For the main analysis, a Cross Lagged Panel Model (CLPM; Fig. 1) was tested to examine the longitudinal, bidirectional effects of father engagement, father parenting aggravation, and child behavior in predicting delinquency over the course of four time points spanning 12 years (age 3 to age 15).

Prior to analyses, the sample (*N* = 3,296) for the current study was restricted to families with at least one valid father report across the included waves. Analyses were conducted in Mplus V8.8 with Full Information Maximum Likelihood Estimation with robust standard errors to account for missing data by using all available observations, as well as non-normally distributed variables, while minimizing bias and preserving the full sample size (Graham et al., 2013; Wothke, 2000). A Random Intercept Cross-Lagged Panel Model (RI-CLPM) was initially considered to separate within- and between-person effects (Hamaker et al., 2015); however, the model did not converge despite multiple specifications. Further, following recommendations that RI-CLPMs are not appropriate for models with limited time points and large gaps between waves (Hamaker, 2026; Orth et al., 2021), the more parsimonious cross-lagged panel model was utilized. The reciprocal relationships among the variables across the four timepoints were estimated, including both autoregressive and cross-lagged paths in order to account for not only the stability of variables but also the possible bidirectional associations over time.

Time-varying covariates (mother engagement, mother aggravation in parenting, and parental relationship status) were included in the analyses as predictors at each included wave, such that their effects were estimated concurrently with the focal study variables across development. In addition, several time-invariant covariates (family poverty-to-income ratio, father education, father race, and child sex) were included to account for stable background characteristics at age 3, with all model paths adjusted for their effects. Model fit was evaluated with comparative fit index (CFI) and root mean square error of approximation (RMSEA), with CFIs equal or greater than 0.95 and RMSEA equal or less than 0.06 indicating good model fit (Hu & Bentler, 1999). Given the large sample size, alpha was set at *p* < .01 for all tests of statistical significance.

The hypothesized indirect effects were tested using bias-corrected bootstrapping with 5,000 bootstrap samples. Sex differences in the focal pathways were tested with multigroup models. Equality constraints were imposed on autoregressive and focal cross-lagged paths across sex. Time-varying maternal covariates and demographic covariates were included in all models but were not constrained across groups. Then, the fit of the constrained model was compared to the fit of an unconstrained model where all paths were allowed to vary by sex. As the main models utilized the MLR estimator, the Satorra-Bentler correction (Satorra & Bentler, 2010) was applied to all likelihood ratio tests of group differences for the multigroup analyses. Better fit of the unconstrained model indicated the presence of sex differences and was followed by comparing the constrained model to models with one path freed at a time to identify paths that vary by child sex.

## Results

### Preliminary Results

Descriptive statistics are reported in Table 1, with bivariate correlations between main study variables and time-varying covariates reported in Table 2. Several significant correlations emerged among the main study variables and the time-invariant demographic variables. Both adolescents and primary caregivers reported higher problem behaviors, including externalizing and delinquency measures, among males across waves, *r*s=-0.07 to − 0.20, all *p*<.001. Further, fathers reported less engagement with girls, *r*=-.06, *p*=.002, compared to boys. Compared to White fathers, Hispanic fathers reported higher aggravation in parenting and lower father engagement at child age 9 (*r*s = 0.06 to.08, *p*s < 0.01). Having a Hispanic father was also correlated with lower levels of child delinquency at age 9 (*r* = − .14, *p* < .001), though not age 15, and lower primary caregiver-reported child externalizing behaviors at both ages 9 and 15 (*r*s = − 0.07, *p*s < 0.001), compared to White fathers. In contrast, having a Black father was associated with higher levels of child delinquency and primary caregiver-reported externalizing behaviors (*r*s = 0.06 to 0.19, all *p*s < 0.01) across all waves, as well as lower aggravation in parenting (*r* = − .07, *p* < .001), compared to White fathers. No correlations emerged between Black father status and father engagement. Further, father higher education is associated with lower primary caregiver-reported child externalizing behavior problems at all waves, *r*s=-0.10 to − 0.15, *p*<.001, as well as lower delinquency at age 9, *r*=-.09, *p*<.001, and age 15, *r*=-.10, *p*<.001.

**Table 1:** Descriptive Statistics

	Mean	SD	%	Range
Delinquency (Age 9)	1.24	1.77		0–17
Delinquency (Age 15)	1.08	1.64		0–12
Child Externalizing Behavior (Age 3)	0.61	0.36		0-1.95
Child Externalizing Behavior (Age 5)	0.42	0.96		0-1.5
Child Externalizing Behavior (Age 9)	0.22	0.22		0–2
Child Externalizing Behavior (Age 15)	0.22	0.25		0-1.8
Father Engagement (Age 3)	4.53	1.08		0–7
Father Engagement (Age 5)	3.96	1.27		0–7
Father Engagement (Age 9)	2.14	0.86		0–4
Father Aggravation (Age 3)	2.94	0.66		1–4
Father Aggravation (Age 5)	2.99	0.69		1–4
Father Aggravation (Age 9)	3.14	0.68		1–4
Mother Engagement (Age 3)	5.00	0.90		0–7
Mother Engagement (Age 5)	4.65	1.16		0–7
Mother Engagement (Age 9)	3.74	0.57		1–5
Mother Aggravation (Age 3)	2.25	0.67		1–4
Mother Aggravation (Age 5)	2.18	0.68		1–4
Mother Aggravation (Age 9)	2.03	0.68		1–4
Parent Relationship Status (Age 3)			57.04%	0–1
Parent Relationship Status (Age 5)			47.47%	0–1
Parent Relationship Status (Age 9)			41.30%	0–1
Parent Relationship Status (Age 15)			32.23%	0–1
Child Sex (Female)			47.80%	1–2
Father Race (Black)			47.04%	0–1
Father Race (Hispanic)			26.97%	0–1
Father Education (Higher Ed)			40.71%	0–1
Family Income to Poverty Ratio (Age 15)	2.40	2.66		0-41.89

**Table 2:** Correlations among Main Variables and Time-Varying Covariates

	1.	2.	3.	4.	5.	6.	7.	8.	9.	10.	11.	12.	13.	14.	15.	16.	17.	18.	19.	20.
1. F Engage (A3)	-																			
2.F Aggravation (A3)	.08**	-																		
3.C Behavior (A3)	.01	− .08*	-																	
4. M Engage (A3)	.16**	.05	− .09**	-																
5. M Aggravation (A3)	− .08**	− .18**	.25**	− .16**	-															
6. P Relation (A3)	− .02	− .01	− .11**	.00	− .06**	-														
7. F Engage (A5)	.55**	.11**	− .03	.14**	− .14**	− .20	-													
8. F Aggravation (A5)	.09**	.60**	− .06	.03	− .12**	.02	.14**	-												
9. C Behavior (A5)	− .02	− .10**	.56**	− .09**	.19**	− .13**	− .02	− .10**	-											
10. M Engage (A5)	.11**	.03	− .08**	.51**	− .14**	− .03	.18**	− .01	− .06*	-										
11.M Aggravation (A5)	− .08**	− .16**	.22**	− .11**	.58**	− .05*	− .08**	− .14**	.28**	− .15**	-									
12. P Relation (A5)	− .03	.03	− .08**	.01	− .04	.72**	.09*	.05	− .13**	.01	− .07**	-								
13. F Engage (A9)	.27**	.06	− .06	.05	− .03	.32**	.30**	.14**	− .10**	.03	− .04	.36**	-							
14.F Aggravation (A9)	.07*	.41**	− .09**	.02	− .09**	.09**	.06	.50**	− .10**	− .03	− .07*	.11**	.11**	-						
15. C Behavior (A9)	.02	− .00	.40**	− .03	.14**	− .11**	− .01	− .05	.51**	− .01	.20**	− .11**	− .08*	− .11**	-					
16. Delinquency (A9)	− .01	− .04	.21**	− .01	.05*	− .10**	.02	− .04	.23**	.02	.07**	− .10**	− .02	− .09**	.31**	-				
17. M Engage (A9)	.12**	− .02	− .03	.33**	− .05*	− .05*	.10**	− .01	− .03	.38**	− .03	− .05*	.10**	− .02	− .00	.05*	-			
18. M Aggravation (A9)	− .03	− .09**	.22**	− .10**	.44**	− .07**	− .04	− .10**	.22**	− .09**	.51**	− .09**	− .04	− .14**	.25**	.08**	− .06**	-		
19. P Relation (A9)	− .04	.03	.09**	− .00	− .03	.58**	.02	.04	− .16**	− .03	− .06**	.71**	.40**	.08**	− .13**	− .13**	− .06*	− .08**	-	
20.C Behavior (A15)	− .01	− .04	.30**	− .06*	.12**	− .13**	− .00	− .06	.42**	− .03	.15**	− .15**	− .13**	− .14**	.52**	.29**	− .03	.19**	− .16**	-
21. Delinquency (A15)	.03	− .05	.09**	− .04	.06*	− .12**	.02	− .05	.20**	− .02	.06*	− .11**	− .02	− .08*	.20**	.25**	− .02	.07**	− .13**	.33**

Note: 1=Father Engagement Age 3, 2=Father Aggravation Age 3, 3= Child Behavior Problems Age 3, 4=Mother Engagement Age 3, 5=Mother Aggravation Age 3, 6=Parent Relationship Status Age 3, 7=Father Engagement Age 5, 8=Father Aggravation Age 5, 9= Child Behavior Problems Age 5, 10=Mother Engagement Age 5, 11=Mother Aggravation Age 5, 12=Parent Relationship Status Age 5, 13=Father Engagement Age 9, 14=Father Aggravation Age 9, 15= Child Behavior Problems Age 9, 16=Delinquency Age 9, 17=Mother Engagement Age 9, 18=Mother Aggravation Age 9, 19= Parent Relationship Status Age 9, 20=Child Behavior Problems Age 15, 21=Teen Delinquency Age 15.p<.01*p<.001**

Missing data analyses using logistic regression indicated that participants not retained by age 15 did not differ on any study variables from those with complete data across waves, including main variables of interest and covariates.

### Main Analyses

#### Age 3 to Age 5

Standardized parameter estimates are included in Table 3, with effects of covariates reported in Supplemental Materials. The cross-lagged panel model had good model fit, CFI=0.968, RMSEA=0.036, CI_90%_[0.034; 0.039]). Father aggravation in parenting was associated with subsequent levels of father engagement with small effect sizes. Specifically, higher parenting aggravation at child age 3 was associated with higher engagement at age 5, opposite to expectations. However, father aggravation in parenting at age 3 was not associated with subsequent child externalizing behavior problems. Father engagement at age 3 was not related to subsequent father aggravation in parenting nor child externalizing behavior problems at age 5. Child externalizing behavior problems at age 3 were not associated with father parenting aggravation or engagement at age 5, indicating a lack of evocative effects.

**Table 3:** Standardized Coefficients for Age 3 Variables Predicting Age 5 Outcomes from the Cross-Lagged Panel Model

	Age 5				
	Father Engagement	Father Aggravation	Child Behavior	Mother Engagement	Mother Aggravation
**Age 3**	**β (SE** **)**	**β (SE)**	**β (SE)**	**β (SE)**	**β (SE)**
Father Engagement	.54 (.02)**	.05 (.02)	− .02 (.02)	.03 (.02)	− .03 (.02)
Father Aggravation	.06 (.02) *	.59 (.02)**	− .03 (.03)	− .00 (.02)	− .04 (.02)
Child Behavior	− .01 (.03)	− .01 (.03)	.52 (.02)**	− .03 (.02)	.07 (.02)**
*Covariates*					
Mother Engagement	.05 (.02)	− .01 (.02)	− .02 (.02)	.52 (.02)**	− .01 (.02)
Mother Aggravation	− .02 (.02)	− .02 (.02)	.03 (.02)	− .05 (.02)*	.56 (.02)**
Parent Relationship Status	.08 (.03)*	.02 (.03)	− .08 (.02)**	− .03 (.02)	− .02 (.02)
Child Sex (Female)	− .01 (.02)	− .01 (.02)	− .03 (.02)	.02 (.02)	− .01 (.02)
Father Race/Ethnicity (Black)	− .04 (.03)	− .02 (.02)	− .00 (.02)	− .01 (.02)	− .02 (.02)
Father Race/Ethnicity (Hispanic)	− .04 (.02)	.01 (.02)	.01 (.03)	− .08 (.02)**	− .01 (.02)
Father Education (Higher Ed)	− .01 (.02)	.03 (.02)	− .05 (.02)	− .03 (.02)	− .05 (.02)
Family Income-to-Poverty Ratio	− .04 (.03)	− .00 (.02)	− .04 (.02)	− .01 (.02)	.02 (.02)

*Italic* values indicated autoregressive paths; **Bolded** values indicate statistical significance at *p*<.01

Note: p<.01*, p<.001**

#### Age 5 to Age 9

Standardized parameter estimates are included in Table 4. At age 5, father aggravation in parenting was not associated with subsequent father engagement, primary caregiver reported child externalizing behavior problems, nor child reported delinquency. Similarly, father engagement at age 5 was not related to subsequent father parenting aggravation, primary caregiver reported child externalizing behavior problems, nor child self-reported delinquency at age 9. Primary caregiver reported child externalizing behaviors at age 5 were significantly related to child-self reported delinquency at age 9. However, no evocative effects of primary caregiver reported child externalizing behavior problems on father aggravation in parenting nor father engagement emerged.

**Table 4:** Standardized Coefficients for Age 5 Variables Predicting Age 9 Outcomes from the Cross-Lagged Panel Model

	Age 9					
	Father Engagement	Father Aggravation	Child Behavior	Delinquency	Mother Engagement	Mother Aggravation
**Age 5**	**β (SE)**	**β (SE)**	**β (SE)**	**β (SE)**	**β (SE)**	**β (SE)**
Father Engagement	.30 (.03)**	− .00 (.03)	.01 (.02)	− .01 (.03)	.05 (.03)	.00 (.02)
Father Aggravation	.05 (.03)	.50 (.02)**	.03 (.02)	− .01 (.02)	− .00 (.02)	− .02 (.02)
Child Behavior	− .03 (.03)	− .04 (.03)	.49 (.03)**	.19 (.03)**	− .03 (.02)	.10 (.02)**
*Covariates*						
Mother Engagement	− .02 (.02)	− .02 (.02)	.00 (.02)	.02 (.02)	.37 (.02)**	− .05 (.02)
Mother Aggravation	.01 (.04)	.02 (.02)	.07 (.02)*	.01 (.02)	.01 (.02)	.47 (.02)**
Parent Relationship Status	.32 (.02)**	.07 (.02)*	− .03 (.02)	− .01 (.02)	− .02 (.02)	− .02 (.02)
Child Sex (Female)	− .06 (.03)*	.01 (.02)	− .06 (.02)*	− .19 (.02)**	.04 (.02)	.01 (.02)
Father Race/Ethnicity (Black)	− .06 (.03)	.02 (.02)	− .03 (.02)	.15 (.02)**	.07 (.03)*	.01 (.02)
Father Race/ Ethnicity (Hispanic)	− .03 (.03)	.07 (.02)*	− .09 (.03)**	− .04 (.02)	− .05 (.03)	− .06 (.02)*
Father Education (High School)	.06 (.03)	.00 (.02)	− .04 (.03)	− .06 (.02)*	− .02 (.02)	− .03 (.02)
Family Income-to-Poverty Ratio	.06 (.02)**	.04 (.02)	− .04 (.02)*	.02 (.02)	− .04 (.02)	− .00 (.02)

*Italic* values indicated autoregressive paths; **Bolded** values indicate statistical significance at *p*<.01

#### Age 9 to Age 15

Standardized parameter estimates are included in Table 5. At age 9, neither father engagement nor father aggravation in parenting was associated with primary caregiver-reported child externalizing behavior problems or teen self-reported delinquency at age 15. Higher primary caregiver reported child externalizing behaviors at age 9 were related to higher teen self-reported delinquency at age 15, and higher self-reported delinquency at age 9 was related to higher subsequent primary caregiver reported child externalizing behavior problems at age 15.

**Table 5:** Standardized Coefficients for Age 9 variables predicting Age 15 outcomes from the Cross-Lagged Panel Model

	Age 15	
	Child Behavior	Delinquency
**Age 9**	**β (** ***SE*** **)**	**β (** ***SE*** **)**
Father Engagement	− .04 (.03)	.05 (.03)
Father Aggravation	− .05 (.02)	− .04 (.02)
Child Behavior	.45 (.03)**	.12 (.03)**
Delinquency	.13 (.02)**	.16 (.03)**
*Covariates*		
Mother Engagement	− .02 (.02)	− .05 (.02)
Mother Aggravation	.06 (.02)*	.01 (.02)
Parent Relationship Status	− .03 (.02)	− .07 (.02)*
Child Sex (Female)	.02 (.02)	− .07 (.02)*
Father Race/Ethnicity (Black)	.01 (.02)	.11 (.03)**
Father Race/Ethnicity (Hispanic)	− .02 (.02)	.07 (.03) *
Father Education (Higher Ed)	− .06 (.02)*	− .03 (.02)
Family Income-to-Poverty Ratio	− .02 (.02)	− .01 (.02)

*Italic* values indicated autoregressive paths; **Bolded** values indicate statistical significance at *p*<.01

### Indirect Effects

No significant indirect effects emerged, indicating a lack of support for mediation. Father aggravation in parenting at age 3 was not indirectly related to later delinquency at age 15 through greater early externalizing behaviors at ages 5 and 9, β=-0.002, *p*=.174, CI99%[-0.016, 0.003], nor was it related indirectly through lower father engagement at ages 5 and 9, β = 0.001, *p*=.203 CI99%[-0.001, 0.007]. Further, child externalizing behavior problems at age 3 were not associated with higher father parenting aggravation at age 5, which was not, in turn, related to lower levels of father engagement at age 9, β=-0.001, *p*=.644 CI99%[-0.014, 0.006]. Lastly, father engagement at age 5 was not indirectly related to teen delinquency, β = 0.001, *p*=.450, CI99%[-0.008, 0.011], nor child externalizing behavior problems, β = 0.004, *p*=.748, CI99%[-0.005, 0.006], at age 15 through child externalizing behavior problems at age 9.

### Multigroup Moderation

Using Likelihood Ratio Testing with the Satorra-Bentler correction (Satorra & Bentler, 2010), the results indicated that the constrained model did not fit significantly worse than the freely estimated multigroup model, Δχ^2^ (28) = 30.60, *p* = .335, indicating that the paths between father engagement, father aggravation in parenting, child externalizing behaviors, and later teen delinquency were not moderated by child sex. Paths for the freely estimated multigroup model are shown in supplemental materials.

## Discussion

Although prior research has linked negative child outcomes to lower father engagement (Sarkadi et al., 2008) and high aggravation in parenting (Bronte-Tinkew et al., 2010), few studies have examined how father engagement and aggravation in parenting are reciprocally associated with externalizing behaviors, which may spill over into adolescent delinquency. Grounded in a Developmental Cascade framework, this study used a four-wave longitudinal design to examine the bidirectional relationships between father engagement, father aggravation in parenting, and child behavior problems in childhood, and their links to delinquency in adolescence, addressing the relative lack of father-focused longitudinal research on developmental risk processes (Schulz et al., 2023). Contrary to expectations, results from this study revealed limited longitudinal associations and no evidence for indirect pathways to adolescent delinquency. The lack of indirect effects is notable, given that one of the primary goals of the study was to test whether father engagement, father aggravation in parenting, and child behavior processes contributed indirectly to adolescent delinquency. These findings suggest that the links between early fathering processes and adolescent delinquency may be more complex, potentially depending on contextual factors or developmental processes not captured in this study.

Early child externalizing behavior problems emerged as the most consistent and robust predictor of adolescent delinquency. Consistent with previous research (e.g., Anderson et al., 2024; Assink et al., 2015), these findings underscore the importance of early behavior problems for later adjustment. In contrast, no evidence of father engagement or aggravation in parenting predicting adolescent delinquency was found. These null findings may suggest that, within this sample and developmental period, father engagement and aggravation in parenting are not robust predictors of child externalizing behavior problems or delinquency, once stability in child behavior and broader family and demographic factors are taken into account. Additionally, the use of multiple reporters across constructs, such as fathers reporting on engagement and parenting aggravation, mothers reporting on child externalizing behaviors, and adolescents reporting on their own delinquency, may have introduced reporter-specific biases and cross-informant discrepancies that weakened longitudinal associations. Given previous research identifying fathering characteristics as important predictors of teen delinquency, the absence of father-driven effects was unexpected. The null findings may suggest that other paternal characteristics, such as fathers’ psychopathology (e.g., Gulenc et al., 2018), paternal warmth (Rothenberg et al., 2020), or criminal behaviors (e.g., Porter & King, 2015), may be more salient predictors of child externalizing behavior problems and teen adolescent delinquency than fathers’ engagement or aggravation in parenting.

Contrary to expectations, there was no evidence of evocative effects of child externalizing behavior problems on father engagement or father aggravation in parenting, with findings providing little evidence of longitudinal associations in which child externalizing behavior problems predict subsequent reports of father engagement and aggravation in parenting. It is possible that other child characteristics, such as child temperament, are more salient in affecting fathers’ stress and behaviors (Oddi et al., 2013). Given the mixed findings regarding child evocative effects on fathers, additional longitudinal research is needed to clarify which child characteristics are most influential and under what developmental or contextual conditions such effects emerge.

Unexpectedly, higher father aggravation in parenting at child age 3 was associated with higher father engagement at age 5. This pattern contrasts with previous findings suggesting that parenting stress reduces involvement (Ward & Lee, 2020). One possible explanation is that greater engagement itself creates more opportunities for parenting aggravation, such that fathers who are more involved may also be more likely to experience frustration in their parenting role. Further, at age 3, both father engagement and aggravation in parenting were positively correlated. In other words, higher father engagement may co-occur with greater frustration in their parenting role, suggesting that subsequent aggravation may be partly a byproduct of increased contact and involvement. Thus, rather than reflecting a reciprocal cycle where parenting aggravation reduces engagement, the results may indicate that aggravation in the parenting role may reflect fathers’ investment and concern about their role as a parent. Alternatively, the unexpected association may reflect measurement differences or other unmeasured variables, such as child temperament or personality traits eliciting both greater engagement and parenting-related distress. However, as this was a rather small effect, this unexpected finding needs to be replicated before it can be fully interpreted or understood.

Surprisingly, there was no evidence of sex differences in the longitudinal associations between father engagement, father aggravation in parenting, child externalizing behavior problems, and adolescent delinquency. Although boys typically exhibit higher levels of externalizing behaviors and delinquency than girls (Fairchild & Smaragdi, 2018) and fathers may differ in their patterns of engagement with sons versus daughters (Ogg & Anthony, 2019), the developmental pathways linking these constructs appeared to be comparable across child sex in the present study. These findings are consistent with prior research indicating that adolescent sex did not moderate associations between fathers’ positive engagement and externalizing behaviors (Temmen & Crockett, 2021), as well as research showing no evidence of sex differences in the pathways linking paternal incarceration, father engagement, child behavior problems, and delinquency (Anderson et al., 2024). Together, these findings suggest that although boys and girls may differ in their average levels of externalizing behavior problems, delinquency, and certain parenting experiences, the underlying longitudinal processes connecting father engagement and aggravation in parenting to child behavioral adjustment may operate similarly across sexes. However, given the limited and mixed literature on sex differences in father-child developmental processes, these findings should be interpreted cautiously and replicated in future research.

Although the present study focused on the role of fathers, it is notable that maternal aggravation in parenting showed more consistent associations with their reports of child externalizing behavior problems, with findings suggesting a more consistent pattern of transactional associations between maternal aggravation and child externalizing behaviors than was observed for fathers. Mothers in the current study were considered to be primary caregivers, which may be indicative of more frequent opportunities to shape, and be shaped, by their children’s behaviors over time. Importantly, though, maternal aggravation in parenting was not associated with adolescent self-reported delinquency, suggesting that the maternal effects may be more closely tied to their perception, not necessarily child behavior itself. Thus, the stronger associations observed for maternal variables should not necessarily be interpreted as evidence that mothers are more influential than fathers. Nevertheless, these findings highlight the importance of considering parenting aggravation in the emergence and development of problem behaviors in children and adolescence.

### Implications

Findings from this study highlight the enduring role of early child externalizing behaviors in predicting later delinquency, underscoring the importance of early intervention and prevention efforts aimed at addressing externalizing behaviors during childhood. The lack of consistent longitudinal associations involving father aggravation in parenting and father engagement raises important questions about how these fathering experiences function over time, perhaps suggesting that their roles may operate in more nuanced or conditional ways. The associations may also be shaped by broader ecological stressors, like economic strain or neighborhood disadvantage, and by dynamics within the family system, such as coparenting quality (e.g., Bronte-Tinkew et al., 2010) and the interdependence between mothers, fathers, and children, emphasizing the importance of continuing to consider the impact of fathers in these broader contexts. Further, given the positive correlation between father engagement and aggravation in the parenting role, results suggest that policies promoting father engagement (e.g., paternity leave, father-friendly programming) and programs aimed at educating fathers about the parenting role (e.g., McGinnis et al., 2019) should consider that increased engagement may co-occur with aggravation in the parenting role. Attending to this co-occurrence may be important for maximizing long-term benefits of engagement-focused initiatives. Finally, children’s perceptions of fathering may be more strongly tied to outcomes than fathers’ self-reports, underscoring the importance of incorporating multiple informants in research and practice.

### Strengths, Limitations, and Future Directions

The present study included several strengths, such as the inclusion of a four-wave longitudinal design over sensitive periods of development using robust methodology. One of the major limitations of the study involved the measurement of father and mother engagement, as the measures only encompassed a small number of activities, as well as only probing for frequency of behaviors. Additionally, differences in the reference periods of the engagement measures may have introduced measurement inconsistency across time, potentially limiting strict comparability of levels across assessments. Given this, the measures of engagement used in the current project may not be sufficiently nuanced to capture meaningful variations in engagement behaviors. More robust measures of parent engagement should be included in future research to advance our understanding of the role of parental engagement in child development by probing not only the frequency of engagement, but also the quality of the engaged behaviors. Moreover, although developmentally appropriate at each wave, the operationalization of engagement shifted as children matured, potentially altering the construct’s meaning across time and limiting strict longitudinal comparability. Further, the current study was unable to probe for child’s perceptions of their father’s engagement and aggravation in parenting, which may be more salient to the development of externalizing behaviors and delinquency. Future research should consider potential discrepancies between father and child perceptions by utilizing a multi-informant approach.

Additionally, the current study was unable to estimate a Random-Intercept cross-lagged panel model due to non-convergence issues. Future studies should include more frequent measurements and limit large gaps between waves to be able to employ RI-CLPM to separate between- and within-person differences. Another limitation of the current study is that several measures demonstrated relatively low internal consistency, which may have introduced measurement errors and attenuated associations. Future research should aim to replicate these findings using more reliable or multi-method approaches to strengthen confidence in the observed effects. Future research should continue to investigate the role of fathers within a developmental cascade framework but broaden the scope of father characteristics examined to include risk factors such as psychopathology (e.g., Gulenc et al., 2018), criminal involvement (e.g., Porter & King, 2015), harsh or abusive parenting (Pinquart, 2021), paternal warmth (e.g., Rothenberg et al., 2020), and socioeconomic stressors (e.g., Dwyer Emory, 2018). Lastly, future studies should explicitly model the interplay between maternal and paternal engagement and aggravation in parenting, as such approaches may better capture the distinct and potentially interactive pathways through which parenting processes are linked to externalizing behavior problems and delinquency.

## Conclusion

Despite growing recognition of fathers’ importance in child development, relatively little longitudinal research has examined how father engagement, father aggravation in parenting, and child externalizing behavior problems operate together across childhood to shape adolescent delinquency. To address this gap, this study provides a much-needed longitudinal examination of the reciprocal relationships among fathers’ engagement, aggravation in parenting, and children’s externalizing behaviors and the development of adolescent delinquency. Results suggested that early child externalizing behavior problems were the most consistent predictors of adolescent delinquency, whereas father engagement and father aggravation in parenting demonstrated limited longitudinal associations with externalizing behaviors and adolescent delinquency. These results highlight both the enduring influence of children’s early externalizing behaviors and the complexity of father–child dynamics across development. By focusing on fathers, an often underrepresented group in developmental research, this work contributes to a more inclusive understanding of family influences on youth outcomes and underscores the central role of early externalizing behaviors in shaping adolescent delinquency.

## Supplementary Information

Below is the link to the electronic supplementary material.

Supplementary Material 1

## Data Availability

The FFCWS data are publicly available at https://ffcws.princeton.edu/. The analytic code and output are available upon request.

## Declarations

### Preregistration

This study was not pre-registered.

## References

[CR1] Abidin, R. R. (1983). *Parenting Stress Index: Manual, Administration Booklet, [and] Research Update*. Pediatric Psychology.

[CR2] Achenbach, T. M. (1992). *Manual for the Child Behavior Checklist / 2–3 and 1992 Profile*. University of Vermont Department of Psychiatry.

[CR4] Achenbach, T. M., & Rescorla, L. A. (2000). *Manual for the ASEBA preschool forms and profiles* (Vol. 30). University of Vermont, Research center for children, youth, & families.

[CR3] Achenbach, T. M., Ivanova, M. Y., Rescorla, L. A., Turner, L. V., & Althoff, R. R. (2016). Internalizing/Externalizing Problems: Review and Recommendations for Clinical and Research Applications. *Journal of the American Academy of Child & Adolescent Psychiatry*, *55*(8), 647–656. 10.1016/j.jaac.2016.05.012 27453078

[CR5] Anderson, A. J., Henrich, C. C., & Mrug, S. (2024). Paternal Incarceration and Adolescent Delinquency: Role of Father Engagement and Early Child Behavior Problems. *Prevention Science*. 10.1007/s11121-024-01734-2 PMC11652568 39356351

[CR6] Annunziato, R. A., Rakotomihamina, V., & Rubacka, J. (2007). Examining the Effects of Maternal Chronic Illness on Child Well-Being in Single Parent Families. *Journal of Developmental & Behavioral Pediatrics*, *28*(5), 386. 10.1097/DBP.0b013e3181132074 18049321

[CR7] Assink, M., van der Put, C. E., Hoeve, M., de Vries, S. L. A., Stams, G. J. J. M., & Oort, F. J. (2015). Risk factors for persistent delinquent behavior among juveniles: A meta-analytic review. *Clinical Psychology Review*, *42*, 47–61. 10.1016/j.cpr.2015.08.002 26301752

[CR8] Awada, S. R., Corning, E. R., & Shelleby, E. C. (2020). Maternal experiences of intimate partner violence and adolescent delinquency: Pathways through parenting stress and harsh parenting behaviors. *Journal of Family Trauma Child Custody & Child Development*, *17*(3), 268–292. 10.1080/26904586.2020.1806771

[CR9] Barbot, B., & Hunter, S. R. (2012). Developmental Changes in Adolescence and Risks for Delinquency. In E. L. Grigorenko (Ed.), *Handbook of Juvenile Forensic Psychology and Psychiatry* (pp. 11–34). Springer US. 10.1007/978-1-4614-0905-2_2

[CR10] Barroso, N. E., Mendez, L., Graziano, P. A., & Bagner, D. M. (2018). Parenting Stress through the Lens of Different Clinical Groups: A Systematic Review & Meta-Analysis. *Journal of Abnormal Child Psychology*, *46*(3), 449–461. 10.1007/s10802-017-0313-6 PMC5725271 28555335

[CR11] Bronte-Tinkew, J., Horowitz, A., & Carrano, J. (2010). Aggravation and Stress in Parenting: Associations with Coparenting and Father Engagement Among Resident Fathers. *Journal of Family Issues*, *31*(4), 525–555. 10.1177/0192513X09340147

[CR12] Bureau of Justice Statistics (2024). Juvenile Court Statistics 2022 Report *| Office of Juvenile Justice and Delinquency Prevention*. (2024, December 27). https://ojjdp.ojp.gov/statistical-briefing-book/access-juvenile-court-statistics-2022-report

[CR13] Carlson, M. J., & McLanahan, S. S. (2010). Fathers in fragile families. In M. E. Lamb (Ed.), *The role of the father in child development* (5th ed., pp. 241–269). John Wiley & Sons, Inc.

[CR14] Carter, A. (2019). The Consequences of Adolescent Delinquent Behavior for Adult Employment Outcomes. *Journal of Youth and Adolescence*, *48*(1), 17–29. 10.1007/s10964-018-0934-2 30298224

[CR15] Cheung, S., & Huang, C. C. (2024). Adolescent delinquency: The role of early childhood exposure to intimate partner violence. *Family Relations*, *73*(2), 874–890. 10.1111/fare.12949

[CR16] Choy, O., Raine, A., Venables, P. H., & Farrington, D. P. (2017). Explaining the Gender Gap in Crime: The Role of Heart Rate. *Criminology*, *55*(2), 465–487. 10.1111/1745-9125.12138

[CR17] Climent-Galarza, S., Alcaide, M., Garcia, O. F., Chen, F., & Garcia, F. (2022). Parental Socialization, Delinquency during Adolescence and Adjustment in Adolescents and Adult Children. *Behavioral Sciences*, *12*(11). 10.3390/bs12110448 PMC9687913 36421744

[CR18] Cobb-Clark, D. A., & Tekin, E. (2011). *Fathers and Youth’s Delinquent Behavior* (Working Paper 17507). National Bureau of Economic Research. 10.3386/w17507

[CR19] Cox, M. J., & Paley, B. (1997). Families as Systems. *Annual Review of Psychology*, *48*(Volume 48, 1997), 243–267. 10.1146/annurev.psych.48.1.243 9046561

[CR20] Deater-Deckard, K. (1998). Parenting Stress and Child Adjustment: Some Old Hypotheses and New Questions. *Clinical Psychology: Science and Practice*, *5*(3), 314–332. 10.1111/j.1468-2850.1998.tb00152.x

[CR21] Deater-Deckard, K., & Panneton, R. (2017). Unearthing the developmental and intergenerational dynamics of stress in parent and child functioning. In K. Deater-Deckard & R. Panneton (Eds.), *Parental stress and early child development: Adaptive and maladaptive outcomes* (pp. 1–11). Springer International Publishing/Springer Nature. 10.1007/978-3-319-55376-4_1

[CR22] Drury, A. J., DeLisi, M., & Elbert, M. J. (2020). What Becomes of Chronic Juvenile Delinquents? Multifinality at Midlife. *Youth Violence and Juvenile Justice*, *18*(2), 119–134. 10.1177/1541204019858741

[CR23] Dwyer Emory, A. (2018). Explaining the Consequences of Paternal Incarceration for Children’s Behavioral Problems. *Family Relations*, *67*(2), 302–319. 10.1111/fare.12301

[CR24] Easterbrooks, M. A., Raskin, M., & McBrian, S. F. (2014). Father Involvement and Toddlers’ Behavior Regulation: Evidence from a High Social Risk Sample. Fathering, 12(1), 71–93. https://www.proquest.com/docview/1640746332/abstract/459AFB0F69E24FD7PQ/1

[CR26] Fagan, J., & Lee, Y. (2014). Longitudinal Associations among Fathers’ Perception of Coparenting, Partner Relationship Quality, and Paternal Stress during Early Childhood. *Family Process*, *53*(1), 80–96. 10.1111/famp.12055 24236848

[CR25] Fagan, J., Bernd, E., & Whiteman, V. (2007). Adolescent Fathers’ Parenting Stress, Social Support, and Involvement with Infants. *Journal of Research on Adolescence*, *17*(1), 1–22. 10.1111/j.1532-7795.2007.00510.x

[CR27] Fairchild, G., & Smaragdi, A. (2018). The Neurobiology of Offending Behavior in Adolescence. In The Wiley Blackwell Handbook of Forensic Neuroscience (pp. 421–453). 10.1002/9781118650868.ch16

[CR28] Fang, Y., Luo, J., Boele, M., Windhorst, D., van Grieken, A., & Raat, H. (2024). Parent, child, and situational factors associated with parenting stress: A systematic review. *European Child & Adolescent Psychiatry*, *33*(6), 1687–1705. 10.1007/s00787-022-02027-1 PMC11211171 35876894

[CR29] Flouri, E., Midouhas, E., & Narayanan, M. K. (2016). The Relationship Between Father Involvement and Child Problem Behaviour in Intact Families: A 7-Year Cross-Lagged Study. *Journal of Abnormal Child Psychology*, *44*(5), 1011–1021. 10.1007/s10802-015-0077-9 26349744

[CR30] *Future of Families and Child Wellbeing Study (FFCWS)*. (n.d.). Retrieved September 2, (2025). from https://ffcws.princeton.edu/data-and-documentation/public-data-documentation

[CR31] Gajos, J. M., Leban, L., Weymouth, B. B., & Cropsey, K. L. (2023). Sex Differences in the Relationship Between Early Adverse Childhood Experiences, Delinquency, and Substance Use Initiation in High-Risk Adolescents. *Journal of Interpersonal Violence*, *38*(1–2), 311–335. 10.1177/08862605221081927 35466765

[CR32] Gargano, L. M., Locke, S., Li, J., & Farfel, M. R. (2018). Behavior problems in adolescence and subsequent mental health in early adulthood: Results from the World Trade Center Health Registry Cohort. *Pediatric Research*, *84*(2), 205–209. 10.1038/s41390-018-0050-8 PMC6185774 29907850

[CR33] Graham, J. W., Cumsille, P. E., & Shevock, A. E. (2013). Methods for handling missing data. In W. F. V. J. A. Schinka, & I. B. Weiner (Eds.), *Handbook of psychology: Research methods in psychology* (pp. 109–141). Wiley.

[CR34] Gryczkowski, M. R., Jordan, S. S., & Mercer, S. H. (2010). Differential Relations between Mothers’ and Fathers’ Parenting Practices and Child Externalizing Behavior. *Journal of Child and Family Studies*, *19*(5), 539–546. 10.1007/s10826-009-9326-2

[CR35] Gulenc, A., Butler, E., Sarkadi, A., & Hiscock, H. (2018). Paternal psychological distress, parenting, and child behaviour: A population based, cross-sectional study. *Child: Care Health and Development*, *44*(6), 892–900. 10.1111/cch.12607 30155910

[CR36] Hamaker, E. L. (2026). The within-between dispute in cross-lagged panel research and how to move forward. *Psychological Methods*. 10.1037/met0000600 37902677

[CR37] Hamaker, E. L., Kuiper, R. M., & Grasman, R. P. P. P. (2015). A critique of the cross-lagged panel model. *Psychological Methods*, *20*(1), 102–116. 10.1037/a0038889 25822208

[CR38] Harris, K. M., & Udry, J. R. (2018). National Longitudinal Study of Adolescent to Adult Health (Add Health), 1994– 2008 [Public Use]. Carolina Population Center, University of North Carolina-Chapel Hill [distributor], Interuniversity Consortium for Political and Social Research [distributor]. 10.3886/ICPSR21600.v21

[CR39] Hu, L., & Bentler, P. M. (1999). Cutoff criteria for fit indexes in covariance structure analysis: Conventional criteria versus new alternatives. *Structural Equation Modeling: A Multidisciplinary Journal*, *6*(1), 1–55. 10.1080/10705519909540118

[CR40] Isen, J., Tuvblad, C., Younan, D., Ericson, M., Raine, A., & Baker, L. A. (2022). Developmental Trajectories of Delinquent and Aggressive Behavior: Evidence for Differential Heritability. *Child Psychiatry & Human Development*, *53*(2), 199–211. 10.1007/s10578-020-01119-w PMC8280243 33449264

[CR41] Jeynes, W. H. (2015). A Meta-Analysis: The Relationship Between Father Involvement and Student Academic Achievement. *Urban Education*, *50*(4), 387–423. 10.1177/0042085914525789

[CR42] Jia-Richards, M., Versace, A., Bachrach, R. L., & Wang, F. L. (2025). Facets of trait impulsivity and their relationships to developmental trajectories of externalizing behaviors from childhood into early adolescence. *Journal of Research in Personality*, *114*, 104555. 10.1016/j.jrp.2024.104555 PMC13132145 42078474

[CR43] Jiang, Q., Wang, D., Yang, Z., & Choi, J. K. (2023). Bidirectional relationships between parenting stress and child behavior problems in multi-stressed, single-mother families: A cross-lagged panel model. *Family Process*, *62*(2), 671–686. 10.1111/famp.12796 PMC10411985 35667879

[CR44] Kennedy, L. A., & Gelman, A. (2018). Year 15 Fragile Families survey weight adjustment.

[CR45] Kim, J., Kim, R., Oh, H., Lippert, A. M., & Subramanian, S. V. (2020). Estimating the influence of adolescent delinquent behavior on adult health using sibling fixed effects. *Social Science & Medicine*, *265*, 113397. 10.1016/j.socscimed.2020.113397 33010637

[CR46] Kochanova, K, Pittman, L. D., & McNeela, L. (2022). Parenting Stress and Child Externalizing and Internalizing Problems Among Low-Income Families: Exploring Transactional Associations. *Child Psychiatry & Human Development*, 53(1), 76–88. . 10.1007/s10578-020-01115-0. 33398689

[CR47] Lamb, M. E. (2000). The History of Research on Father Involvement. *Marriage & Family Review*, *29*(2–3), 23–42. 10.1300/J002v29n02_03

[CR48] Lee, J. (2021). First-Year maternal employment, resources, and trajectories of father engagement with preschoolers. *Early Child Development and Care*, *191*(11), 1692–1706. 10.1080/03004430.2019.1671833

[CR49] Leve, L. D., & Cicchetti, D. (2016). Longitudinal transactional models of development and psychopathology. *Development and Psychopathology*, *28*(3), 621–622. 10.1017/S0954579416000201 27427795

[CR50] Masten, A. S., & Cicchetti, D. (2010). Developmental cascades. *Development and Psychopathology*, *22*(3), 491–495. 10.1017/S0954579410000222 20576173

[CR51] Maumary-Gremaud, A. (2000). Things that you have done: Fast track project technical report. *Durham, NC: Fast Track Data Center, Conduct Problems Preventions Research Group*.

[CR52] McDaniel, B. T., & Radesky, J. S. (2020). Longitudinal Associations Between Early Childhood Externalizing Behavior, Parenting Stress, and Child Media Use. *Cyberpsychology Behavior and Social Networking*, *23*(6), 384–391. 10.1089/cyber.2019.0478 PMC7301326 32096655

[CR53] McGinnis, S., Lee, E., Kirkland, K., Smith, C., Miranda-Julian, C., & Greene, R. (2019). Engaging At-Risk Fathers in Home Visiting Services: Effects on Program Retention and Father Involvement. *Child and Adolescent Social Work Journal*, *36*(2), 189–200. 10.1007/s10560-018-0562-4

[CR54] Moffitt, T. E., & Caspi, A. (2001). Childhood predictors differentiate life-course persistent and adolescence-limited antisocial pathways among males and females. *Development and Psychopathology*, *13*(2), 355–375. 10.1017/S0954579401002097 11393651

[CR55] Oddi, K. B., Murdock, K. W., Vadnais, S., Bridgett, D. J., & Gartstein, M. A. (2013). Maternal and Infant Temperament Characteristics as Contributors to Parenting Stress in the First Year Postpartum. *Infant and Child Development*, *22*(6), 553–579. 10.1002/icd.1813

[CR56] Ogg, J., & Anthony, C. J. (2019). Parent involvement and children’s externalizing behavior: Exploring longitudinal bidirectional effects across gender. *Journal of School Psychology*, *73*, 21–40. 10.1016/j.jsp.2019.02.002 30961879

[CR57] Orth, U., Clark, D. A., Donnellan, M. B., & Robins, R. W. (2021). Testing prospective effects in longitudinal research: Comparing seven competing cross-lagged models. *Journal of Personality and Social Psychology*, *120*(4), 1013–1034. 10.1037/pspp0000358 PMC7854859 32730068

[CR58] Paz, Y., Orlitsky, T., Roth-Hanania, R., Zahn-Waxler, C., & Davidov, M. (2021). Predicting externalizing behavior in toddlerhood from early individual differences in empathy. *Journal of Child Psychology and Psychiatry*, *62*(1), 66–74. 10.1111/jcpp.13247 32645218

[CR59] Pei, F., Wang, X., Yoon, S., & Tebben, E. (2019). The influences of neighborhood disorder on early childhood externalizing problems: The roles of parental stress and child physical maltreatment. *Journal of Community Psychology*, *47*(5), 1105–1117. 10.1002/jcop.22174 30811041

[CR60] Pinquart, M. (2021). Cultural Differences in the Association of Harsh Parenting with Internalizing and Externalizing Symptoms: A Meta-Analysis. *Journal of Child and Family Studies*, *30*(12), 2938–2951. 10.1007/s10826-021-02113-z

[CR61] Porter, L. C., & King, R. D. (2015). Absent Fathers or Absent Variables? A New Look at Paternal Incarceration and Delinquency. *Journal of Research in Crime and Delinquency*, *52*(3), 414–443. 10.1177/0022427814552080

[CR62] Puzzanchera, C., Hockenberry, S., & Sickmund, M. (2022). Youth and the juvenile justice system: 2022 national report. https://ojjdp.ojp.gov/library/publications/youth-and-juvenile-justice-system-2022-national-report

[CR63] Reichman, N. E., Teitler, J. O., Garfinkel, I., & McLanahan, S. S. (2001). Fragile Families: Sample and design. *Children and Youth Services Review*, *23*(4–5), 303–326. 10.1016/S0190-7409(01)00141-4

[CR64] Rescorla, L. A. (2005). Assessment of young children using the Achenbach System of Empirically Based Assessment (ASEBA). *Mental Retardation and Developmental Disabilities Research Reviews*, *11*(3), 226–237. 10.1002/mrdd.20071 16161094

[CR65] Rothenberg, W. A., Lansford, J. E., Alampay, L. P., Al-Hassan, S. M., Bacchini, D., Bornstein, M. H., Chang, L., Deater-Deckard, K., Giunta, L. D., Dodge, K. A., Malone, P. S., Oburu, P., Pastorelli, C., Skinner, A. T., Sorbring, E., Steinberg, L., Tapanya, S., Tirado, L., M. U., & Yotanyamaneewong, S. (2020). Examining effects of mother and father warmth and control on child externalizing and internalizing problems from age 8 to 13 in nine countries. *Development and Psychopathology*, *32*(3), 1113–1137. 10.1017/S0954579419001214 PMC7308194 31865926

[CR66] Sarkadi, A., Kristiansson, R., Oberklaid, F., & Bremberg, S. (2008). Fathers’ involvement and children’s developmental outcomes: A systematic review of longitudinal studies. *Acta Paediatrica*, *97*(2), 153–158. 10.1111/j.1651-2227.2007.00572.x 18052995

[CR67] Satorra, A., & Bentler, P. M. (2010). Ensuring Positiveness of the Scaled Difference Chi-square Test Statistic. *Psychometrika*, *75*(2), 243–248. 10.1007/s11336-009-9135-y PMC2905175 20640194

[CR68] Schneider, J. L., & West, K. L. (2025). A matter of time: Developmental cascades for developmental science. *Infant Behavior and Development*, *80*, 102078. 10.1016/j.infbeh.2025.102078 40489911

[CR69] Schulz, W., Hahlweg, K., & Supke, M. (2023). Predictors of Fathers’ Participation in a Longitudinal Psychological Research Study on Child and Adolescent Psychopathology. *Journal of Child and Family Studies*, *32*(6), 1666–1680. 10.1007/s10826-022-02521-9

[CR70] Scott, J. K., Nelson, J. A., & Dix, T. (2018). Interdependence among mothers, fathers, and children from early to middle childhood: Parents’ sensitivity and children’s externalizing behavior. *Developmental Psychology*, *54*(8), 1528–1541. 10.1037/dev0000525 29927264

[CR71] Slaughter, J., & Nagoshi, C. T. (2020). Testing a Contextual Model of Effects of Father Involvement on Child Behaviors. *Child & Adolescent Social Work Journal: C & A*, *37*(5), 547–556. 10.1007/s10560-020-00649-5

[CR72] Stone, L. L., Mares, S. H. W., Otten, R., Engels, R. C. M. E., & Janssens, J. M. A. M. (2016). The Co-Development of Parenting Stress and Childhood Internalizing and Externalizing Problems. *Journal of Psychopathology and Behavioral Assessment*, *38*(1), 76–86. 10.1007/s10862-015-9500-3 PMC4789299 27069304

[CR73] Temmen, C. D., & Crockett, L. J. (2021). The importance of father involvement for adolescent internalizing and externalizing symptoms. *Psychology of Men & Masculinities*, *22*(3), 564–577. 10.1037/men0000322

[CR74] Thompson, R., Tabone, J. K., Litrownik, A. J., Briggs, E. C., Hussey, J. M., English, D. J., & Dubowitz, H. (2011). Early Adolescent Risk Behavior Outcomes of Childhood Externalizing Behavioral Trajectories. *The Journal of Early Adolescence*, *31*(2), 234–257. 10.1177/0272431609361203

[CR75] Walters, G. D. (2013). Delinquency, parental involvement, early adult criminality, and sex: Evidence of moderated mediation. *Journal of Adolescence*, *36*(4), 777–785. 10.1016/j.adolescence.2013.03.011 23623004

[CR76] Wang, J., Donithen, R. W., & Schoppe-Sullivan, S. J. (2022). Fathers and Family Systems. In S. Molloy, P. Azzam, & A. Isacco (Eds.), *Handbook of the Psychology of Fatherhood* (pp. 43–59). Springer International Publishing. 10.1007/978-3-031-14498-1_3

[CR77] Ward, K. P., & Lee, S. J. (2020). Mothers’ and fathers’ parenting stress, responsiveness, and child wellbeing among low-income families. *Children and Youth Services Review*, *116*, 105218. 10.1016/j.childyouth.2020.105218 PMC7425837 32801410

[CR78] Willoughby, T., Heffer, T., Good, M., & Magnacca, C. (2021). Is adolescence a time of heightened risk taking? An overview of types of risk-taking behaviors across age groups. *Developmental Review*, *61*, 100980. 10.1016/j.dr.2021.100980

[CR79] Wothke, W. (2000). Longitudinal and multi-group modeling with missing data. In T. D. Little, K. U. Schnabel, & J. Baumert (Eds.), *Modeling longitudinal and multiple group data: Practical issues, applied approaches and specific examples* (pp. 219–240). Lawrence Erlbaum.

[CR80] Yang, P., Pachman, S. L., Schlomer, G. L., & Edin, K. J. (2024). Direct and Indirect Longitudinal Associations of Mother and Father Engagement in Middle Childhood on Adolescent Externalizing and Internalizing Behaviors. *Journal of Youth and Adolescence*, *53*(8), 1832–1846. 10.1007/s10964-024-01982-z PMC12094873 38600264

[CR81] Yoder, J. R., Brisson, D., & Lopez, A. (2016). Moving Beyond Fatherhood Involvement: The Association Between Father–Child Relationship Quality and Youth Delinquency Trajectories. *Family Relations*, *65*(3), 462–476. 10.1111/fare.12197

[CR82] Yu, S. M., & Singh, G. K. (2012). High Parenting Aggravation Among US Immigrant Families. *American Journal of Public Health*, *102*(11), 2102–2108. 10.2105/AJPH.2012.300698 PMC3477940 22994171

