# Supplementary material for: Transactional Pathways from Early Externalizing Behavior to Adolescent Delinquency: The Roles of Father Engagement and Parenting Aggravation
Source: J Youth Adolesc. 2026 Aug 19;55(10):2617–33. doi: 10.1007/s10964-026-02402-0 (PMC13631029; doi:10.1007/s10964-026-02402-0)
Supplement: Supplementary file 1 [file 10964_2026_2402_MOESM1_ESM.docx]

**Supplemental Results**

**Effects of Covariates**

Higher mother aggravation in parenting at age 3 was related to lower mother engagement at age 5. Additionally, child behavior problems at age 3 were related to subsequent mother aggravation in parenting at age 5. Parents who reported being together at age 3 reported higher subsequent father engagement and lower child behavior problems at age 5. Further, mothers reported being less engaged at age 5 when the father was Hispanic, compared to White fathers.

At age 5, higher mother aggravation in parenting was associated with higher child behavior problems, but not with child self-reported delinquency at age 9. Higher child behavior problems at age 5 were related to higher mother aggravation in parenting at age 9. Parents who were together at age 5 reported higher father engagement and higher father aggravation in parenting. At age 9, fathers reported less engagement with daughters, compared to sons. Girls also had lower child behavior problems and lower self-reported delinquency at child age 9 than boys. Compared to having a White father, having a Black father was associated with higher self-reported delinquency, with mothers reporting more engagement with the child when the father was black. Additionally, Hispanic fathers, compared to white fathers, reported greater aggravation in parenting at age 9, and had children with lower primary-caregiver externalizing behaviors at age 9. Further, mothers reported less aggravation in parenting when the father was Hispanic, compared to white. Fathers’ higher education was associated lower delinquency at age 9. Lower income-to-poverty ratio was associated with lower father engagement and higher child behavior problems at child age 9.

Higher mother aggravation in parenting at age 9 was associated with higher child behavior problems at age 15, but was not related to teen-reported delinquency. Parents who were together at age 9 had children who self-reported lower delinquency at age 15. Girls self-reported lower delinquency at age 15 than boys. Children with Black or Hispanic fathers had higher self-reported delinquency at age 15, compared to those with White fathers. Lastly, higher education in fathers was only associated with lower primary caregiver reported child behavior problems, not teen self-reported delinquency.

Given significant effects involving mother engagement and mother aggravation in parenting as covariates, post-hoc analyses were conducted to evaluate indirect effects associated with these maternal variables, with selected indirect effects aligning with the hypothesized effects for fathers. Similar to results for fathers, no significant indirect effects emerged, indicating a lack of support for mediation. Mother aggravation in parenting at age 3 was not indirectly related to later delinquency at age 15 through greater early problem behaviors at ages 5 and 9, β=.006, *p*=.191, CI99%[-.006, .019], nor was it related indirectly through lower mother engagement at age 5 and 9, β=.000, *p*=.488, CI99%[-.001, .002]. Further, child behavior problems at age 3 were not associated lower levels of mother engagement at age 9 via higher mother parenting aggravation at age 5, β=.001, *p*=.632 CI99%[-.003, .006]. Lastly, mother engagement at age 5 was not indirectly related to teen delinquency, β=.001, *p*=.763, CI99%[-.006, .009], nor child behavior problems, β=.003, *p*=.760 CI99%[-.022, .027], at age 15 through child behavior problems at age 9.

Table S1. Standardized Multigroup Results with main paths freed

|  | Father Engagement (A5) | Father Aggravation (A5) | Child Behavior (A5) | Father Engagement (A9) | Father Aggravation (A9) | Child Behavior (A9) | Delinquency (A9) | Child Behavior (A15) | Delinquency (A15) |
| --- | --- | --- | --- | --- | --- | --- | --- | --- | --- |
|  | β(SE) | β(SE) | β(SE) | β(SE) | β(SE) | β(SE) |  | β(SE) | β(SE) |
| Males |  |  |  |  |  |  |  |  |  |
| Father Engagement (A3) | .51(.03)** | .04(.03) | -.07(.03) |  |  |  |  |  |  |
| Father Aggravation (A3) | .06(.03) | .58(.03)** | -.02(.04) |  |  |  |  |  |  |
| Child Behavior (A3) | -.01(.03) | -.04(.03) | .51(.03)** |  |  |  |  |  |  |
| Father Engagement (A5) |  |  |  | .34(.03)** | -.02(.03) | -.01(.04) | -.01(.03) |  |  |
| Father Aggravation (A5) |  |  |  | .05(.03) | .55(.03)** | .02(.04) | -.02(.03) |  |  |
| Child Behavior (A5) |  |  |  | -.05(.04) | -.07(.03) | .47(.04)** | .20(.04)** |  |  |
| Father Engagement (A9) |  |  |  |  |  |  |  | -.02(.04) | .05(.04) |
| Father Aggravation (A9) |  |  |  |  |  |  |  | -.07(.03) | -.04(.04) |
| Child Behavior (A9) |  |  |  |  |  |  |  | .42(.04)** | .09(.04) |
| Delinquency (A9) |  |  |  |  |  |  |  | .14(.04)** | .16(.04)** |
| Females |  |  |  |  |  |  |  |  |  |
| Father Engagement (A3) | .58(.03)** | .05(.03) | .04 .03) |  |  |  |  |  |  |
| Father Aggravation (A3) | .06(.03) | .59(.03)** | -.05(.03) |  |  |  |  |  |  |
| Child Behavior (A3) | -.01(.03) | .01(.04) | .54(.03)** |  |  |  |  |  |  |
| Father Engagement (A5) |  |  |  | .26(.04)** | .01(.03) | .03(.04) | -.01(.03) |  |  |
| Father Aggravation (A5) |  |  |  | .04(.03) | .46(.03)** | .02(.04) | -.02(.03) |  |  |
| Child Behavior (A5) |  |  |  | -.01(.04) | -.01(.04) | .52(.04)** | .20(.04)** |  |  |
| Father Engagement (A9) |  |  |  |  |  |  |  | -.07(.04) | .04(.04) |
| Father Aggravation (A9) |  |  |  |  |  |  |  | -.02(.03) | -.03(.04) |
| Child Behavior (A9) |  |  |  |  |  |  |  | .51(.03)** | .17(.04)** |
| Delinquency (A9) |  |  |  |  |  |  |  | .13(.04)* | .17(.04)** |
